# Diffusion and surface alloying of gradient nanostructured metals

**DOI:** 10.3762/bjnano.8.59

**Published:** 2017-03-03

**Authors:** Zhenbo Wang, Ke Lu

**Affiliations:** 1 Shenyang National Laboratory for Materials Science, Institute of Metal Research, Chinese Academy of Sciences, 72 Wenhua Road, Shenyang 110016, China

**Keywords:** diffusion, gradient nanostructures, grain boundary, interface, surface alloying

## Abstract

Gradient nanostructures (GNSs) have been optimized in recent years for desired performance. The diffusion behavior in GNS metals is crucial for understanding the diffusion mechanism and relative characteristics of different interfaces that provide fundamental understanding for advancing the traditional surface alloying processes. In this paper, atomic diffusion, reactive diffusion, and surface alloying processes are reviewed for various metals with a preformed GNS surface layer. We emphasize the promoted atomic diffusion and reactive diffusion in the GNS surface layer that are related to a higher interfacial energy state with respect to those in relaxed coarse-grained samples. Accordingly, different surface alloying processes, such as nitriding and chromizing, have been modified significantly, and some diffusion-related properties have been enhanced. Finally, the perspectives on current research in this field are discussed.

## Introduction

A gradient nanostructure is a unique architecture in which a layer of nanoscale-grained structure adheres to a ductile coarse-grained (CG) substrate of the same material; a transition layer with graded grain sizes is positioned between them. This architecture with a graded spatial variation of grain sizes offers a number of unique opportunities to enhance the properties of materials. It enables the delocalization of strains in the nano-grained structures, resulting in plastic deformation behavior fundamentally different from that of the free-standing homogeneous nano-grained and CG samples. For example, a considerable tensile ductility was achieved with the significantly enhanced strength in different metals with a gradient nanostructured (GNS) layer [1–3], distinct from the traditional trade-offs between strength and ductility in conventional metals and alloys. In addition, GNS materials exhibit significantly enhanced wear resistance and fatigue resistance compared with their nano-grained and CG counterparts [4–7].

Investigating the diffusion behavior of GNS materials is an important topic mostly due to the following reasons:

1. Numerous interfaces in the nanostructured layer make it possible to study interfacial diffusion and relative characteristics, such as interfacial structure, precipitation, phase transformation, plastic deformation, and chemical reaction, at lower temperatures. In comparison, it is difficult to obtain enough information on these aspects – particularly on the *C*-type diffusion regime as laid out in [8] – because of an insufficient number of interfaces in CG materials.
2. A graded variation of interface types may accompany the graded distribution of grain sizes in a GNS layer. Such a microstructure provides a unique sample to study the effects of grain size and interface type on diffusion properties.
3. Atomic diffusion is the essential factor in surface alloying processes (such as nitriding and chromizing), and diffusion studies in GNS materials provide insights for modifying traditional surface alloying processes of engineering metallic materials.

GNSs have been generated on various metals by methods with controlled surface plastic deformation [9–11]. In this paper, recent progress in the diffusion studies in GNS metals will be clarified to understand diffusion fundamentals and relationships between microstructure and diffusion in nanostructured materials, followed by some typical applications of GNS on advancing traditional surface alloying processes.

## Review

### Interfacial diffusion in GNS materials

In the 1980s, Gleiter et al. [12–13] successfully synthesized the first nanostructured metallic sample by means of inert gas condensation and consolidation (IGC). Since then, studies have been performed to reveal the diffusion behavior of nanostructured metals worldwide. However, the measured results were rather scattered and even controversial. For instance, markedly promoted diffusion coefficients with a lower activation enthalpy were measured in the nanostructured Cu synthesized by IGC [14–16]. And a comparable diffusivity with that of conventional grain boundaries (GBs) was revealed in a nanostructured γ-Fe–Ni alloy prepared by ball milling with subsequent sintering (BMS), while inter-agglomerate boundaries showed much faster diffusion rates (≈4 orders of magnitude higher) with a lower activation enthalpy than the GB diffusion [17]. Moreover, interfacial diffusion coefficients in an Fe_3_Si nanocomposite produced by crystallization (CRY) from a melt-spun amorphous sample were revealed to be even lower in comparison with those in the CG sample [18].

Particular attention has been paid to the diffusion behavior in nanostructured or ultrafine grained (UFG) materials produced by using severe plastic deformation (SPD), mostly because of the bulk forms of nanostructures. Inconsistences also exist in the experimental results. For example, Würschum et al. [19] found that diffusivities in a SPD Pd sample are similar to those in conventional GBs. However, Kolobov et al. [20] detected an increment of 10^4^–10^5^ in GB diffusivities (with lower activation enthalpy) of Cu in a SPD Ni sample in comparison with those in a CG sample. The diffusion coefficients along some ultra-fast channels (together with some conventional GBs) in an UFG Cu–Zr alloy sample prepared via SPD were found to be ≈10^3^ times higher than those along GBs in a high-purity CG Cu sample [21]. In order to differentiate these ultra-fast diffusion channels from conventional GBs, the term “nonequilibrium” GBs was used. In this case, GBs might gather a large number of irregular (extrinsic) dislocation structures, so that extra free energy might be introduced in the interfaces, while misorientations across them remain stable [22–23]. The existence of nonequilibrium GBs after SPD processing has been supported by strain mapping results in a SPD Pd–Ag alloy, in which the GB thickness (with evident strain) is determined to be ≈1.5 nm and is markedly larger than the thickness of relaxed GBs (≈0.5 nm) [24]. In addition, the higher energy of nonequilibrium GBs as compared to conventional GBs has been demonstrated by the transformation of partial to complete GB wetting in Sn–Pb alloys during continuous strain by Straumal et al. [25]. However, this notion was confused by two works in the SPD Cu and Cu–Pb alloy [26–27], in which various defects such as vacancy agglomerations, nanometer- and micrometer-sized voids were observed. These defects might be linked and significantly accelerate atomic penetration in the materials. In addition, the concurrent influence from lattice diffusion might not be completely excluded from the mean diffusion flux in SPD metals, in which the grain sizes are usually in the sub-micrometer scale (>200 nm) and numerous dislocations exist in grains [28].

The microstructural observations revealed that the GNS layers on metals processed by surface mechanical attrition treatments (SMAT) are porosity-free, and the mean grain sizes are on the nanoscale (<100 nm) in the surface layers with a thickness of tens of micrometers, for example on pure Fe [29] and Cu [30]. In addition, a graded distribution of interfacial structure was obtained in the GNS surface layer of Cu [30], with interfaces sequentially dominated by twin boundaries (TBs), high-angle GBs (HAGBs), low-angle GBs (LAGBs) and dislocation walls with an increasing depth. These microstructures characteristics provided unique opportunities to study the relationship between the diffusion property and microstructure into the nanometer scale. The diffusion studies in these materials will be reviewed and compared with results from other nanostructured metals in this section.

### Diffusion behavior in nanostructured surface layers

**Figure 1:**
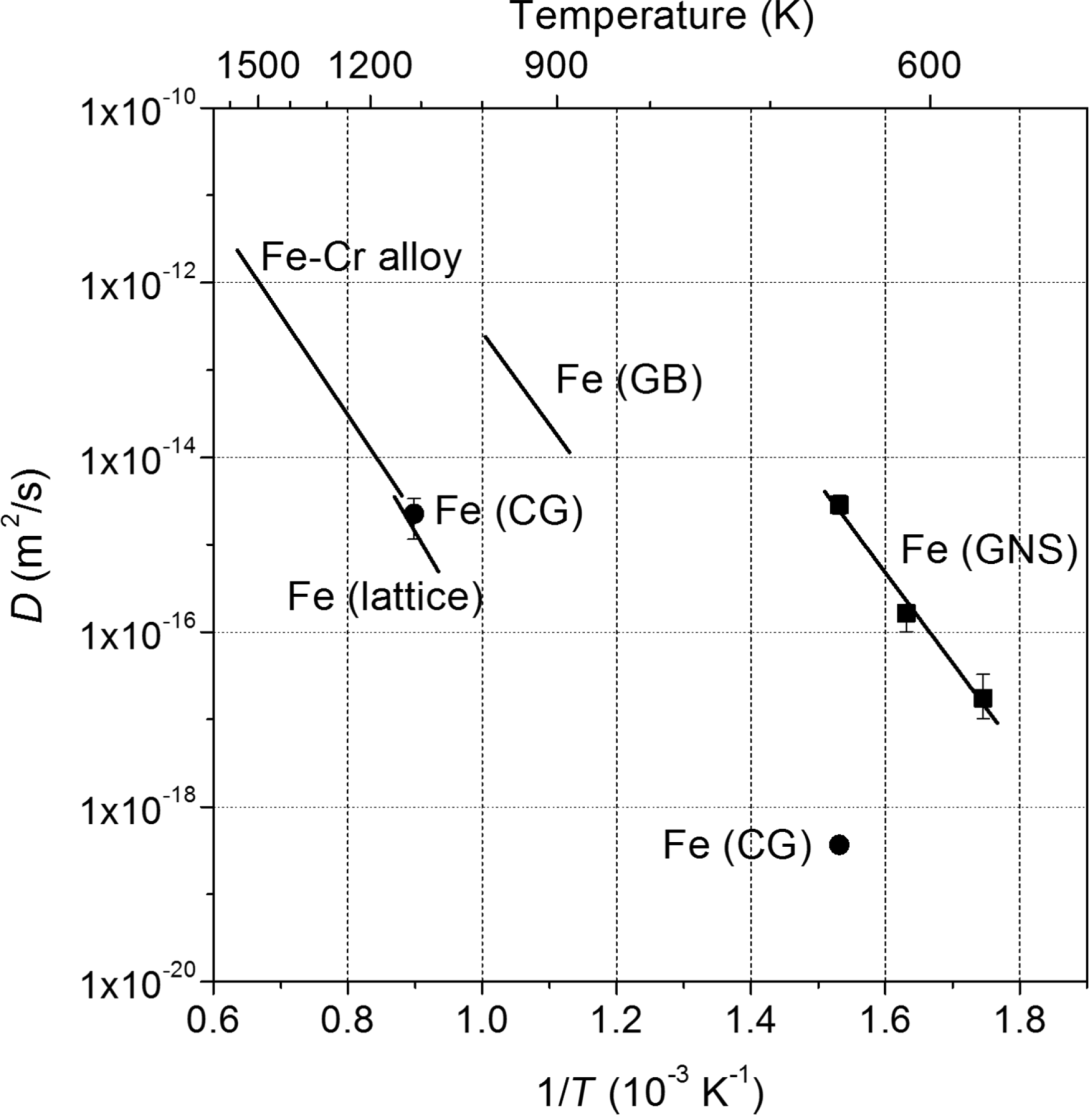
Temperature dependence of the diffusion coefficients of Cr in the GNS Fe produced by SMAT and in the CG Fe, in comparison with those for the α-Fe lattice diffusion, the α-Fe GB diffusion and the diffusion in Fe–Cr alloy. Figure reproduced with permission from [31], copyright 2003 Elsevier.

The diffusion properties of Cr in GNS Fe were measured within the temperature range of 300–380 °C on a SMAT Fe sample with a mean grain size of ≈10 nm in the top surface layer electroplated with a thin layer of Cr [31]. The apparent diffusivity of Cr in the GNS Fe is 7–9 orders of magnitude higher than that in the Fe lattice and 4–5 orders of magnitude higher than that along GBs of ferrite, as shown in Figure 1. In terms of the diffusion kinetics with Arrhenius dependence in the concerned temperature range, the pre-exponential factor and the activation enthalpy of Cr diffusion in the nanostructured Fe were calculated. It is seen that the pre-exponential factor of Cr diffusion in nanostructured Fe is much higher than that for conventional GB diffusion in α-Fe, and the diffusion activation enthalpy is slightly lower than that that of GB diffusion.

**Figure 2:**
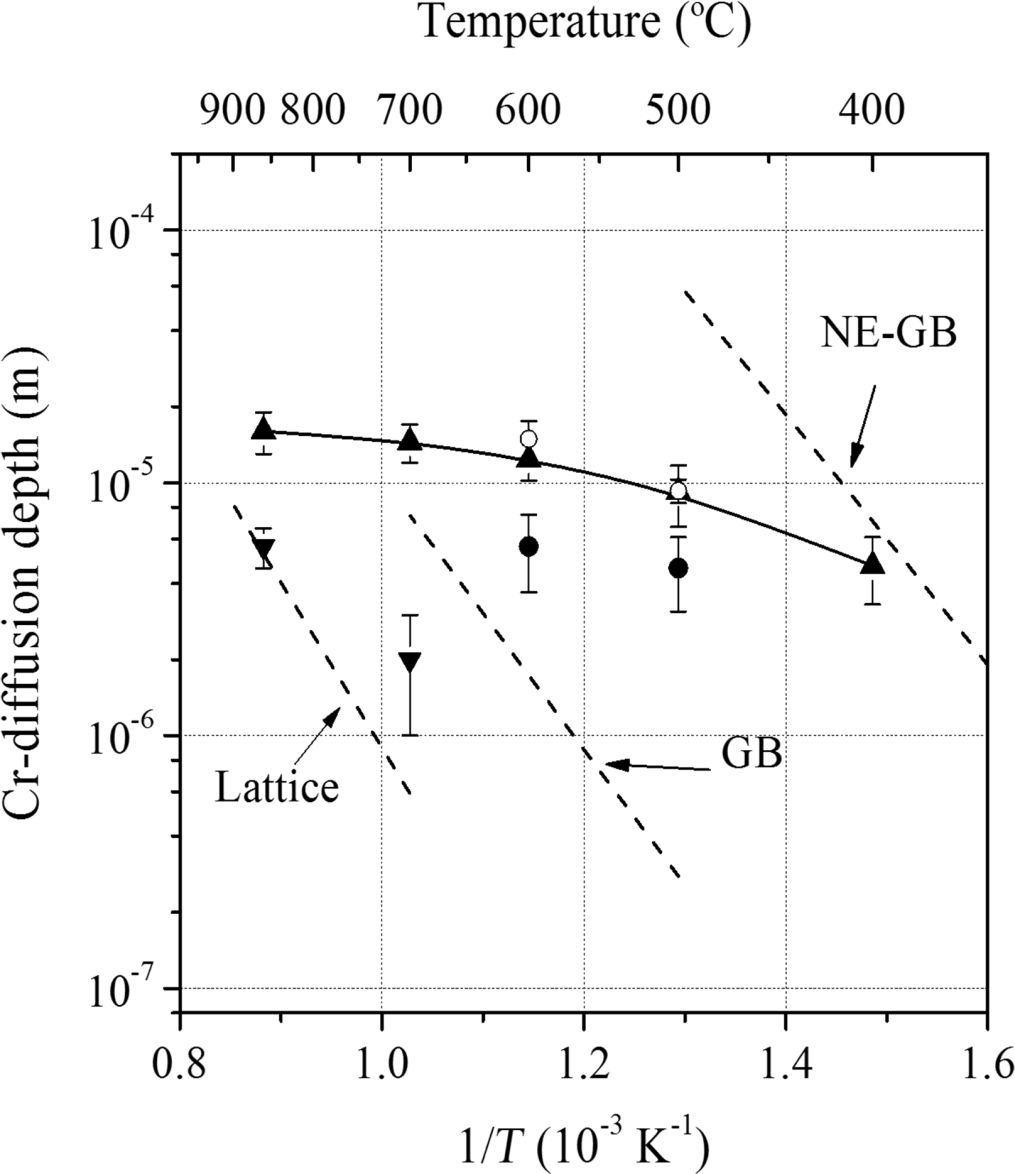
Variations of Cr-diffusion depth with temperature in different low carbon steel samples: as-SMAT (▲), CG (▼), SMAT with subsequent annealing at 300 °C (○) and 400 °C (●) for 180 min. In comparison, the calculation results in terms of the lattice diffusion [34], the conventional GB diffusion (GB) [35], and the diffusion in GNS Fe (NE-GB) [31] were also included. Figure reproduced with permission from [32], copyright 2005 Elsevier.

In addition, the Cr-diffusion depth values were determined in GNS low carbon steel samples chromized in a packed powder mixture of Cr–NH_4_Cl–Al_2_O_3_, as shown in Figure 2 [32]. One can see that the diffusion depth into the as-SMAT sample was larger than that into the CG sample after the same chromizing treatment within the temperature range of 400–860 °C. The ratio of the value into the as-SMAT sample relative to the value into the CG sample decreased with elevating temperatures. A similar trend has also been observed in a GNS H13 steel sample, in which a significantly increased Cr-diffusion depth with respect to that in the CG sample was achieved at chromizing temperatures below 600 °C, and the depth even decreased with increasing temperatures above 600 °C [33]. Meanwhile, it was noticed that an isothermal preannealing might decrease the Cr-diffusion depth. For example, the pre-annealing at 400 °C decreased the Cr-diffusion depth values to ≈60% of the as-prepared GNS low carbon steel (also see Figure 2).

Since the surface Cr concentration is approximately constant during chromizing, the apparent diffusivity in the GNS surface layer (*D*_GNS_) can be calculated from the measured Cr-diffusion depth (*x*) from

[1]
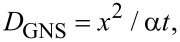


where α is a constant and *t* is the chromizing duration [36]. The results show that *D*_GNS_ is about 8.5 × 10^3^ times higher than the diffusivity along GBs in the CG low carbon steel sample (*D*_RGB_) at 400 °C, and the ratio of *D*_GNS_ to *D*_RGB_ decreases gradually with increasing temperature. While evident grain growth occurred in the nanostructured surface layer, the ratio of *D*_GNS_ to *D*_RGB_ is even less than 1. After the preannealing treatment at 400 °C, *D*_GNS_ decreases to ≈1/4 of that in the as-prepared sample.

Comparing the diffusion studies in GNS surface layers prepared by SMAT with the results in other nanostructured or UFG metals, as in Table 1, we may conclude:

1) The measured interfacial diffusivity is sensitive to the sample preparation method. Diffusivities in nanostructured samples produced by using SMAT or SPD reach a value of 10^3^–10^5^ times higher than those for GBs in CG samples. The diffusivity increment is typically within 10^2^–10^4^ in nanostructured metals produced via the IGC or BMS techniques. Additionally, the interfacial diffusivity in nanostructured samples produced via CRY is much lower than that along GBs in the CG form. Meanwhile, the interfacial diffusivities in nanostructured metals in the as-prepared states decrease significantly after preannealing treatments during which GBs may be relaxed, especially when evident grain growth has occurred.

2) The diffusion activation enthalpies in nanostructured metals are typically 0.5–0.9 of those along GBs in CG samples when no distinct relaxation or grain growth occurs. Additionally, no dependence on the preparation method or grain size is indicated.

3) The effect of grain size on diffusivity seems to be rather weak within the scale regime from 10 to 300 nm. Therefore, the contribution from triple junctions, of which the amount increases with decreased grain size and the diffusivity should be further enhanced due to the larger excess volume [31,37], might be insignificant within the present grain size scale.

**Table 1:** Diffusion studies in nanostructured metals prepared by different techniques.

Material (diffusant)	Grain size (nm)	Preparation method^a^	Diffusion temperature (°C)	*D*_NS_/*D*_RGB_^b^	Arrhenius parameters^c^		Ref.
					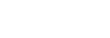	*Q*_NS_/*Q*_RGB_	

Fe (Cr)	10–25	SMAT	300 340 380	2.0 × 10^4^ 9.4 × 10^3^ 1.2 × 10^4^	7 × 10^2^	0.9	[31]
Low-C-steel (Cr)	10–30	SMAT	400 500 600 700	8.5 × 10^3^ 2.1 × 10^2^ 7.9^*^ 0.49^*^	NA	NA	[32]
Low-C-steel (Cr)	10–30	SMAT+PA	500 600	53 1.6^*^	NA	NA	[32]
H13-steel (Cr)	10−30	SMAT	550 600 650	17 11^*^ 0.28^*^	NA	NA	[33]
Cu (Ni)	100	SMAT	130	1.1 × 10^4^	NA	NA	[38]
Ni (Cu)	300	ECAP	125 175	1.1 × 10^5^ 1.0 × 10^4^	4.4 × 10^−11^	0.34	[20]
Pd (Fe)	80–150	SPD	98 200 300	3.0 × 10^3^ 11^*^ 0.12^*^	NA	NA	[19]
Cu–Zr (Ni)	300	ECAP	151–280	0.7–1.4^*^ (0.2–1.3) × 10^3^	0.2 2.3 × 10^3^	0.93 ^s-GB^ 1.06 ^f-GB^	[21]
Cu (Ni)	300	ECAP	151–201	0.5–1.5^*^	0.24	0.94	[39]
Ni (Ni)	300	ECAP	58–127 167–227	(0.7–28) × 10^5^ (0.2–4.5) × 10^3^	6.8 × 10^−4^	0.52	[40]
Cu (Cu)	8	IGC	120	1.1 × 10^2^	3 × 10^−4^	0.59	[14,41]
Cu (Ag)	8	IGC	30–70 80–100	≈2.3 × 10^3^ ≈4.9 × 10^2^	5.9 × 10^−3^ 1.8 × 10^2^	0.53 0.86	[15]
Cu (Au)	10	IGC	100	1.85 × 10^4^	NA	NA	[16]
Pd (Fe)	20	IGC	200 250	7^*^ 0.7^*^	NA	NA	[19]
Cu (Zn)	120	DPD	85–100 115–190	11 0.05–3.5^*^	NA	NA	[42]
γ-FeNi (Ag)	100	BMS	411–547	≈1 (0.34–3.7) × 10^4^	≈1 2 × 10^−3^	≈1 0.53 ^AI^	[17]
Fe_3_Si (Fe)	12	CRY	380–420	≈4 × 10^−5^	NA	1.12 ^a-IF^	[18]

^a^Various preparation methods: SMAT+PA denotes SMAT with subsequent preannealing at 400 °C, when no evident grain growth occurs but microstrain decreases markedly [32]. Equal channel angular pressing (ECAP) is a representative SPD method. DPD denotes the dynamic plastic deformation technique. Other abbreviations are referred to in the main text. ^b^Asterisked values indicate samples with remarkable grain growth during the concerned diffusion annealing. The thickness of GB (δ) was taken as 0.5 nm to calculate *D*_RGB_ when a triple product *s*δ*D*_RGB_ was reported. ^c^Arrhenius parameters: 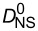 and 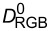 denote the apparent pre-exponential factors for diffusion along interfaces in a nanostructured metal and the relaxed CG sample, respectively; *Q*_NS_ and *Q*_RGB_ denote the diffusion activation enthalpies along interfaces in the nanostructured metal and the relaxed CG sample, respectively; The superscripts s-GB and f-GB denote “slow GB” and “fast GB”, respectively, in [21]; AI denotes agglomerate interfaces, along which the diffusivity (*D*_a_) was derived from a relationship with the product (*P*_a_) as *P*_a_ = *s*_a_·δ_a_·*D*_a_/λ (referring to [17]); a-IF denotes amorphous interfaces in the nanocrystalline Fe_3_Si prepared by CRY [18].

This evidence suggests that GBs in the GNS layers are associated with a higher stored energy and/or with a higher density of defects compared to the GBs in conventional CG counterparts. According to the vacancy mechanism of GB diffusion [43–44], they may facilitate diffusion by decreasing the vacancy formation energy. For example, the existence of a large fraction of free volume in the GNS layer has been demonstrated by the high intensities of the short- and middle-lifetime components in the results of positron annihilation spectroscopy measurements of the surface layer of the SMAT Fe [45].

While GB defects (i.e., extrinsic GB dislocations) are difficult to be identified in GNS or SPD metals, it is argued that the GB diffusion might also be accelerated by GB thickening, resulting from either the GB prewetting (premelting) or the pseudo-partial GB wetting during sample preparation [46–49]. For example, a Zn-rich wetting layer of 2–10 nm in thickness has been formed along Al/Al GBs in UFG Al–Zn alloys after high pressure torsion at room temperature [47]. Dramatically enhanced GB diffusivities were observed in Cu–Bi and Fe–Si–Zn alloys with prewetted GBs [48–49]. Nevertheless, this notion might safely be excluded from the issues accelerating diffusion in the GNS layers, because no system (see Table 1) contains components which are inclined to form GB complexions connected with GB prewetting or pseudo-partial wetting [46,50].

### Diffusion kinetics and free energy of different interfaces

**Figure 3:**
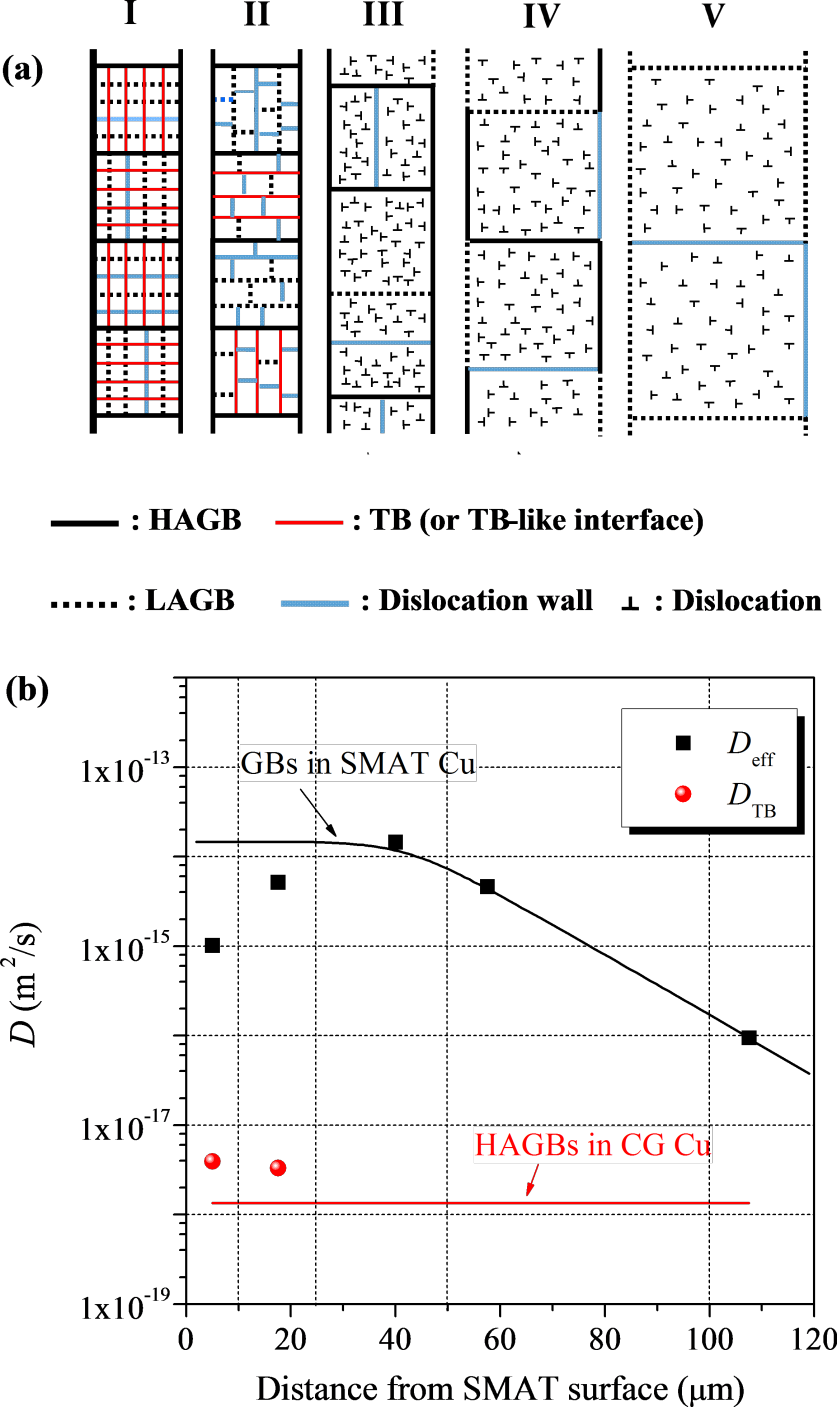
(a) A schematic illustration of the gradient microstructure of grain size and interface in the GNS surface layer of a SMAT Cu sample, and (b) variation in the effective diffusivity of ^63^Ni in different regions at 130 °C. Figure adapted with permission from [38], copyright 2010 Elsevier.

As illustrated in Figure 3a, the grain size increases and interface structure is sequentially dominated by TBs (or TB-like interfaces), HAGBs, LAGBs and dislocation walls with increasing depth in the GNS surface layer on a SMAT pure Cu sample [30,38]. Similar to the accelerated diffusion of Cr observed in GNS Fe and steels, a remarkable penetration of Ni into the GNS surface layer of the SMAT Cu has been detected at temperatures below 150 °C [38,51–52]. The effective diffusivity in the top surface layer is ≈3 orders of magnitude higher than that along relaxed HAGBs in the annealed CG sample at 130 °C. Furthermore, it is noticed that the effective diffusivity in the subsurface layer is higher than that in the top surface layer, and increases with increasing depth up to 30–50 µm, as shown in Figure 3b. After the diffusivity reaches a maximum value of ≈4 orders of magnitude higher than that along the conventional HAGBs, it decreases with further increasing depth.

Since the lattice diffusion distance is negligible at the concerned temperatures, diffusion in the GNS Cu proceeds in accordance to Harrison’s *C–*type diffusion regime [8]. Therefore, the effective diffusivities are directly related to diffusion kinetics along interfaces. As shown in Figure 3b, diffusivities along different interfaces in the GNS surface layer are compared with the diffusivity along HAGBs in a high-purity CG Cu at 130 °C. It is noted that the diffusivities along HAGBs and TBs in the first 25 µm in thickness (i.e., regions I and II) were derived by using the so-called *C*–*B* diffusion mode developed by Divinski et al. [17] in hierarchic microstructures. In this case, the leakage of Ni tracer atoms from HAGBs into TBs (i.e., *B*-regime in the mode) reduces the effective diffusivities in both regions. The diffusivities of HAGBs and TBs in the GNS Cu are about 1.41 × 10^−14^ and 3.65 × 10^−18^ m^2^·s^−1^, respectively.

Table 2 summarizes several works on diffusion along TBs in pure Cu. No comparison with diffusivity along coherent TBs can be made because no diffusion data was provided to the authors’ knowledge. It is shown that the diffusivity along TBs in the SMAT Cu is even slightly higher than the extrapolated diffusivity along GBs in the relaxed CG sample, while those in the DPD and bicrystal samples [42,53] are much lower than the latter. This suggests a much higher diffusivity along TBs in the GNS sample than in other samples. Similar with the situations of GBs (referring to the section “Diffusion behavior in nanostructured surface layers”), the significantly enhanced diffusivity along TBs in the GNS Cu is also expected be induced by a higher stored energy and/or with a higher density of defects. The free energy of GBs varies with the misorientation angle and forms a sharp cusp only several degrees in deviation around the corresponding angle of the coherent TBs, i.e., 70.5° of twin/matrix with [110] symmetric tilt boundary in Cu [54]. In the GNS layer, various dislocations are accumulated at TBs, resulting in a significant increment of interface free energy with only a slight deviation from the ideal TB misorientations [42,54].

**Table 2:** Diffusion data along TBs in different Cu samples.

Base material	Diffusant	Temperature (°C)	*D*_TB_, *P*_TB_^a^ (m^2^·s^−1^, m^3^·s^−1^)	*D*_RGB_, *P*_RGB_^a^ (m^2^·s^−1^, m^3^·s^−1^)	Ref.

SMAT Cu	Ni	130	3.65 × 10^−18^	1.34 × 10^−18^	[38]
DPD Cu	Zn	85 130	6.21 × 10^−20^ 8.61 × 10^−19^	5.82 × 10^−19^ 2.19 × 10^−17^	[42]
Σ3 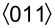 bicrystal Cu	Au	400 500	(0.7–11) × 10^−23^ (0.4–5.8) × 10^−21^	1.18 × 10^−20^ 9.6 × 10^−19^	[53]

^a^Diffusivities were determined in the *C*-type regime in the SMAT and DPD samples. While the values were determined in the *B*-type regime in the bicrystal sample, a triple product *P* was given.

According to a semi-empirical approach developed by Borisov et al. [55], the interface excess free energy difference (Δγ_B_), i.e., the difference between the excess free energy of interfaces in the GNS Cu and that of conventional GBs in a relaxed CG sample at a temperature (*T*), may be estimated from

[2]
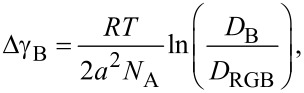


where *a* is the mean distance between atoms at the interface. *N*_A_ and *R* are the Avogadro’s number and gas constant, respectively. *D*_B_ and *D*_RGB_ are the diffusivities of Ni along interfaces in the GNS Cu and along conventional GBs, respectively. By using the diffusion data for different interfaces in the GNS Cu, and *D*_RGB_ from [56], Δγ_B_ values of different interfaces are derived. In comparison with γ_RGB_ (≈0.63 J·m^−2^ [57]), SMAT results in an increment up to 30% in the GB excess free energy in region III, and it decreases with an increasing depth (in regions IV and V). The TB excess free energy in the first 25 µm is comparable with γ_RGB_.

### Reactive diffusion behavior

Reactive diffusion is of great technological importance in the metallurgical industry and in electronic materials. While a reaction process at the interface between two (or more) primary phases usually involves the nucleation and the coarsening stages of the formed phase(s), the nucleation conditions at the interface play decisive roles on the subsequent reactive diffusion process [58].

In comparison with the reactive diffusion processes in CG samples, some previous studies demonstrated different characteristics in nanostructured materials. For instance, Charlot et al. [59] showed that the mechanical milling process applied before the self-propagating high-temperature syntheses (SHS) process markedly decreased the ignition temperature (≈100 °C) of the reaction in Fe and Al powder mixtures, and a nanostructured Fe–Al intermetallic compound was synthesized after the reaction. Meanwhile, more phases from a Ti–Al system were observed on a nanocrystalline Ti substrate with Al layer with respect to a microcrystalline substrate by Wiecinski et al. [60]. The intermetallic layer synthesized on the nanocrystalline Ti substrate was thicker and more homogeneous at 600 °C. Nevertheless, it was noted that grain size effects on reactive diffusion characteristics were still difficult to be understood in these works, mostly because the results were obtained in binary systems with a high heat release by intermetallic formation. SHS occurred at a high temperature and significantly elevated sample temperature after the reaction ignition in such systems [61–62].

In comparison, the enthalpy change and reaction temperature between Fe and Zn (or N) are relatively low, thus SHS will not occur and the nanostructures remain relatively stable during reactive diffusion. In this section, the reactive diffusion behavior of Zn and N into the GNS Fe prepared by SMAT [61–62] will be reviewed from both kinetic and thermodynamic aspects.

### Promoted growth kinetics

The growth kinetics of the Fe–Zn compound layer (mostly ξ-FeZn_13_ phase) was studied in the GNS Fe sample electroplated with a Zn layer at different temperatures from 280 to 340 °C [61]. It was found that the Fe–Zn compound layer formed on the GNS sample is much thicker than that on the CG sample under the same isothermal annealing treatment. According to the relationship between the measured thickness (*l*) of compound layers, diffusion temperature (*T*) and duration (*t*), the growth kinetics was determined from

[3]
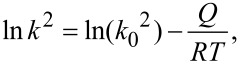


where *k* is the growth constant derived from

[4]
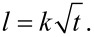


**Figure 4:**
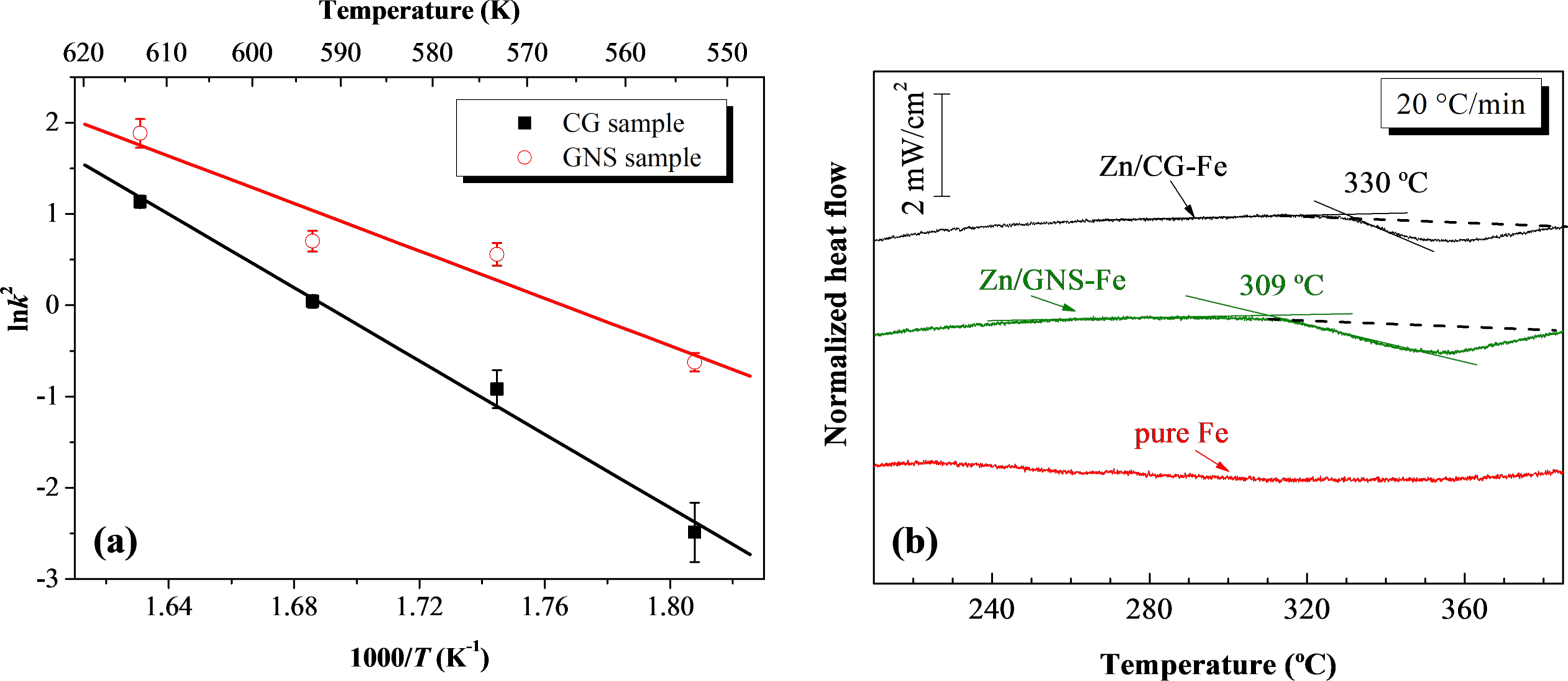
(a) The dependence of the growth constant *k*^2^ on the reciprocal annealing temperature during reactive diffusion of Zn on the GNS and CG Fe samples, and (b) thermal analyses of the Zn/GNS-Fe and the Zn/CG-Fe couples at a heating rate of 20 °C·min^−1^. Figure adapted with permission from [61], copyright 2012 Elsevier.

The pre-exponential factor *k*_0_ is a constant related to atomic diffusion, and *Q* is the activation enthalpy for the growth of compound layer. As derived from Figure 4a, the activation enthalpy for the growth of the compound layer in the GNS sample is 108.0 ± 20.7 kJ·mol^−1^, which is much lower than that in the CG sample (167.1 ± 10.1 kJ·mol^−1^).

Significantly promoted growth kinetics has also been observed in the GNS Fe during gas nitriding at lower temperatures [62–63]. A continuous nitride surface layer (mostly ε-Fe_2–3_N and γ’-Fe_4_N) of ≈10 µm in thickness was formed on the GNS sample after nitriding at 300 °C for 9 h. In comparison, there was no such nitride layer formed on the original CG sample after the same nitriding treatment.

The growth kinetics of the formed compound layer should be controlled by diffusion processes in both the substrates and the compound layer [61]. While the grain size of Fe in the nanostructured surface layer is much smaller than that in the CG matrix, grain sizes of the formed compounds in the GNS sample are still much smaller than those formed in the CG sample [61–62]. Therefore, diffusion of alloying elements in the GNS sample and in the compounds layer is evidently accelerated, and a smaller activation enthalpy is obtained than in the CG sample.

The promoted growth kinetics in GNS samples may also be understood in terms of the reaction kinetics of nucleation and growth. Because GBs usually act as preferable nucleation sites, the existence of a large number of GBs in the nanostructured substrate may result in a significant improvement in the reaction between alloying elements and substrate atoms. The nucleation rate, 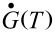, can be written as [64]:

[5]
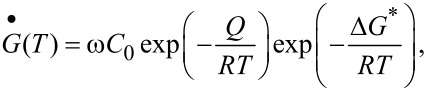


where ΔG^*^ is the critical Gibbs energy for nucleation, ω is a frequency factor, and *C*_0_ is the number of atoms at contacting interfaces. While the value of the critical Gibbs energy for nucleation at GBs is smaller than the value at other regions along the surface, the nucleation rate increases significantly with decreasing grain size in the nanostructured surface layer. The growth kinetics of the nuclei of formed compounds is expected to be significantly promoted by abundant GBs in the GNS layer at the earlier stage of reaction, mostly due to the accelerated diffusion rates of alloying elements along them [32,61].

It is also noted that homogenous reaction layers of FePt and FePt with ≈10 atom % Ag have been synthesized in Pt/Fe and Pt/Ag/Fe thin films, respectively, after annealing at temperatures when the bulk diffusion processes are still frozen [65]. The formation processes have been interpreted as a GB-diffusion-induced solid-state reaction in which the reaction interfaces migrate perpendicularly to the original GB plane, based on corresponding depth profiles of the element concentrations in both systems. This mechanism might also contribute to the promoted growth kinetics of a compound layer in GNS samples.

### Stored energy and reaction thermodynamics

Thermal analysis (see Figure 4b) of the reaction between Zn and GNS Fe showed that an exothermic peak appears at about 309 °C, which is ≈21 °C lower than that in the Zn/CG–Fe couple. Mostly, the ξ-FeZn_13_ phase formed after the exothermic peaks in both samples. In addition, the reaction enthalpy change is ≈58.6 mJ·cm^−2^ in the Zn/GNS–Fe couple, almost double that in the Zn/CG–Fe couple [61].

In addition to the promoted nucleation and growth kinetics related with numerous GBs, as discussed in the last section, the higher stored free energy might also contribute to the lower onset temperature and higher reaction enthalpy change of Fe–Zn reaction in the GNS sample. As we know, stored energy values in metallic systems processed by conventional cold working are typically within 0.01–0.2 kJ·mol^−1^, and they may change either the kinetics or the path of a phase transition in solid-state with a typical free energy change of 0.5–3 kJ·mol^−1^ [66]. While the stored energy may be much higher because GBs are associated with a higher stored free energy in the GNS substrates produced by SMAT [38], an additional driving force will be provided for the formation of intermetallic compounds. For example, the stored energy value in the GNS Fe is estimated to be ≈2.07 kJ·mol^−1^ according to the value in a nanostructured Fe synthesized by ball milling [61,67]. In comparison, the standard Gibbs free energy change for the formation of FeZn_13_ from elemental Fe and Zn atoms is about −2.50 kJ·mol^−1^ at 330 °C [68]. With the stored excess energy in the GNS layer, the Gibbs free energy change for this reaction becomes −4.57 kJ·mol^−1^, so that the nucleation rate will be raised and the onset temperature may be decreased for the Fe–Zn reaction in the Zn/GNS–Fe couple [61].

**Figure 5:**
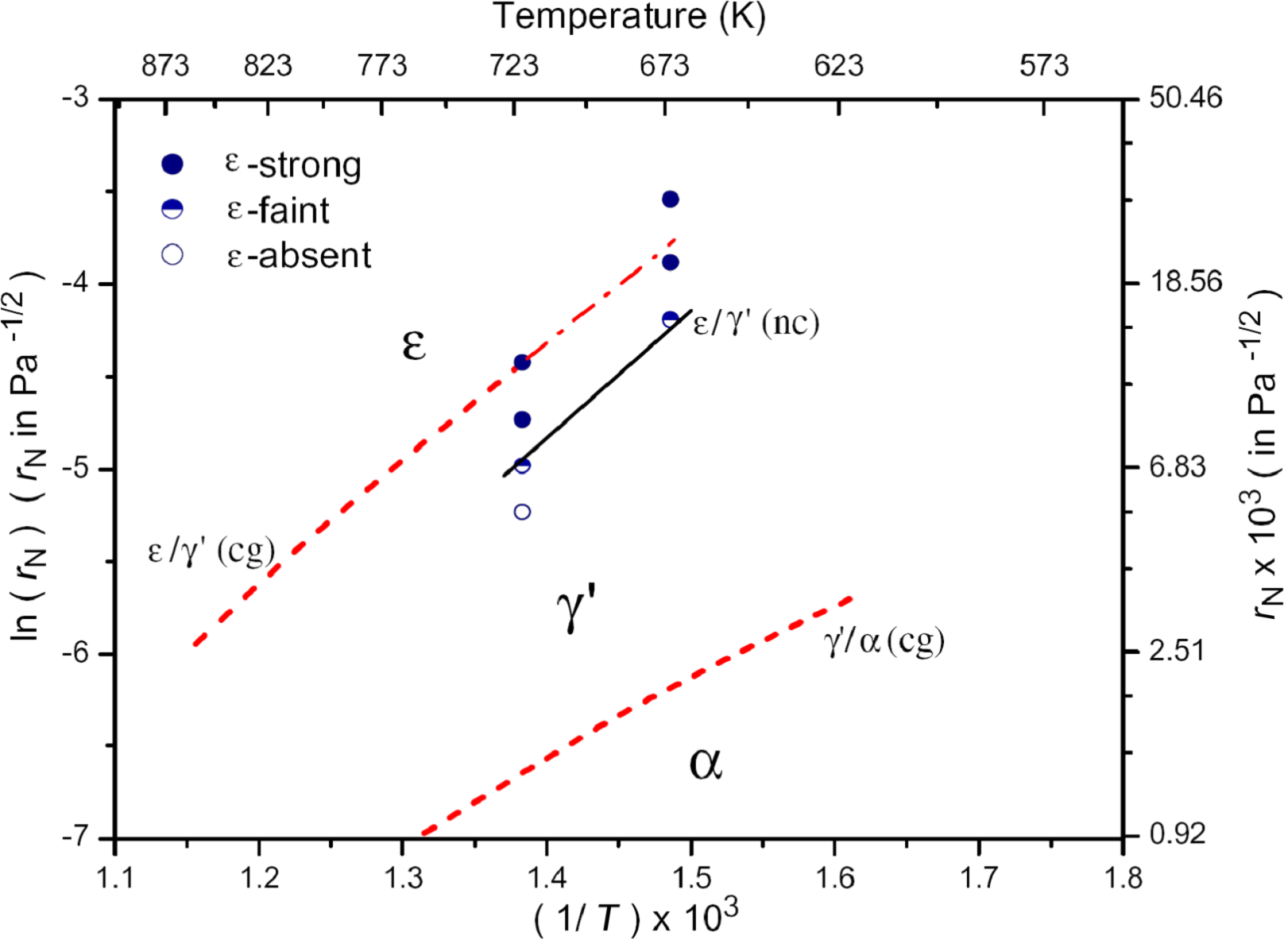
Correlation of temperature and nitrogen potential for the formation of ε-Fe_2–3_N phase in the GNS Fe (nc), in comparison with that for the CG Fe reported by Lehrer [69]. Figure reproduced with permission from [56], copyright 2007 Elsevier.

The stored energy in the nanostructured surface layer also contributed to the promoted formation process of nitrides during nitriding GNS Fe at low temperatures [62]. The Gibbs free energy change for nitrides formation in the CG Fe at 500 °C is about −8.22 kJ·mol^−1^ for the γ’ phase and −1.69 kJ·mol^−1^ for the ε phase. Both values might become positive and nitrides could not form at 300 °C. However, the free energy changes for the nitrides formation became negative with the stored excess energy in the nanostructured Fe phase, so that their formation became possible at the same temperature. The contributions of stored excess energy to the promoted nitriding kinetics have been confirmed by measurements on critical nitrogen potentials for the formation of ε phase. As shown in Figure 5, the ε-Fe_2–3_N phase formed at a lower temperature or with a smaller nitrogen potential in the GNS Fe sample in comparison with those in the CG sample [63,69].

### Surface alloying of GNS materials

Inspired by the significantly promoted diffusion and chemical reaction kinetics in GNS layers, many works have been carried out on advancing surface alloying techniques of metals with a preformed nanostructured surface layer. The advantages are distinct, for example, improving surface properties such as wear and corrosion resistance with higher energy/time efficiency, reducing work-piece distortion and/or grain coarsening. In addition, GNS surface layers with promoted diffusion and chemical reaction were also produced to enhance formation of surface layers with modified microstructure and phase constitutions, in order to be made more protective and functional.

#### Surface alloying processes

**Lower-temperature processes:** Surface alloying processes, such as nitriding and aluminizing, have been achieved on various engineering metals with a GNS surface layer at temperatures much lower than the conventional processing temperatures, and properties such as resistance to wear and corrosion were significantly improved.

**Figure 6:**
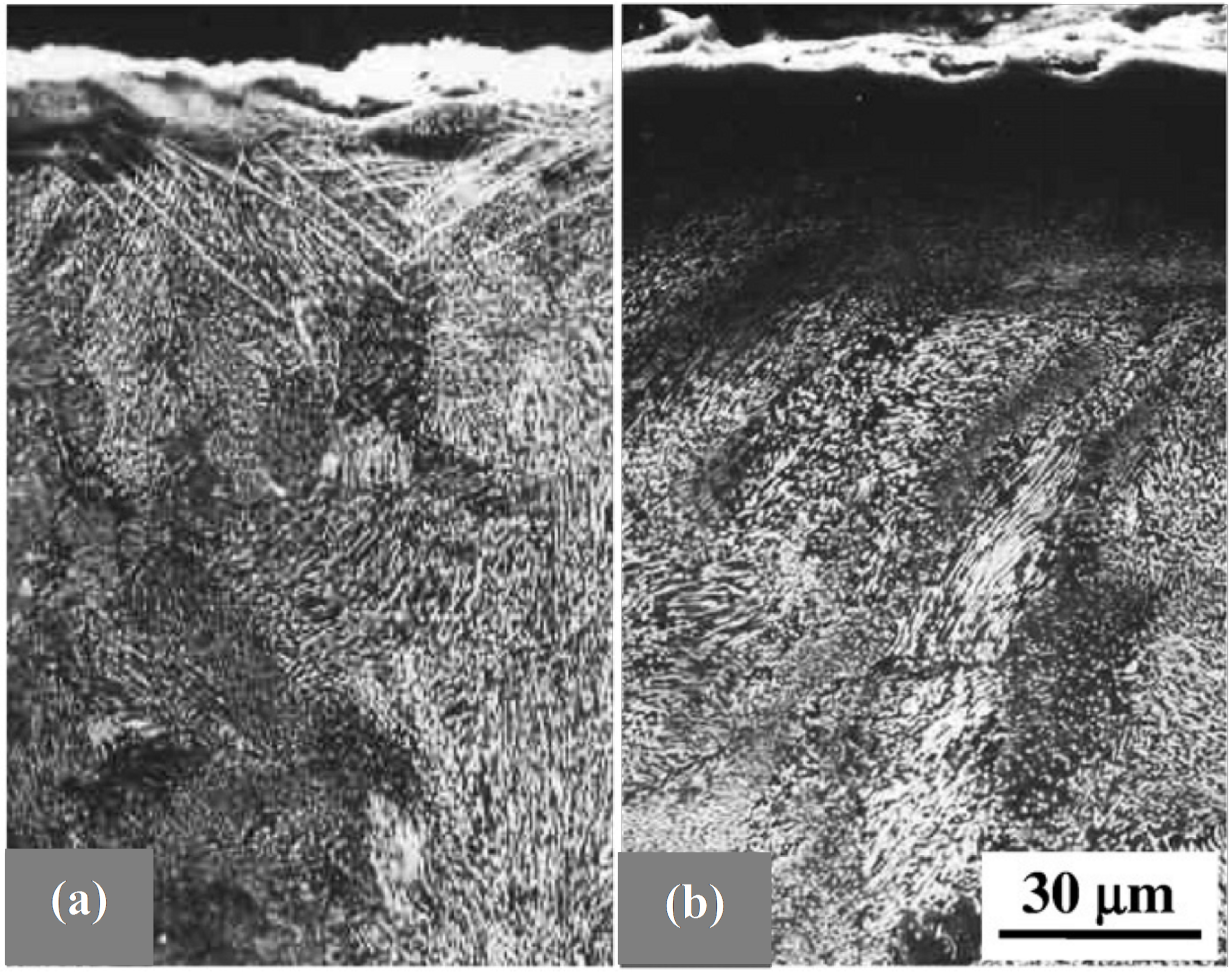
Cross-sectional observations of (a) the CG and (b) the GNS 38CrMoAl samples after gas nitriding at 400 °C for 30 h. Figure reproduced with permission from [70], copyright 2008 Elsevier.

Gas nitriding of steel is conventionally carried out at temperatures above 500 °C over a long duration (60–80 h). A representative nitriding steel 38CrMoAl was submitted to SMAT followed by gas nitriding at 400 °C for 30 h [70]. As shown in Figure 6, a continuous compound layer containing mostly nanosized ε-Fe_2-3_N and γ’-Fe_4_N phases followed by a diffusion zone was formed on the GNS sample, and the total thickness of the compound layer and the diffusion layer is more than 200 μm. In comparison, no continuous compound layer was formed on the CG sample after the same nitriding process, and only a diffusion zone with the thickness of ≈50 μm was observed. Thus, a much higher wear resistance was obtained due to the thicker nitride layer with increased hardness on the GNS sample.

Similarly, preformed GNS surface layers have also been used to successfully lower the boronizing temperature of tool steel [71–75], aluminizing temperature of Mg alloys [76] and ferritic steel [77–78], and nitriding temperature of plain carbon steel [79], stainless steel [80–82], Ti and Ti alloys [83–85], etc.

In addition, the simultaneous surface mechanical and alloying treatment has also been developed to promote the formation of alloyed or compound layers. By loading specimens, pack-aluminizing powder and alloy balls in a chamber, and then mechanically vibrating the chamber at an elevated temperature (≈600 °C), an aluminized coating consisting mostly of Fe_2_Al_5_ phase with some FeAl_3_ and FeAl phases has been produced on low-carbon steel [86] and Fe-13Cr steel [87] by Zhan et al. While grain growth is effectively hindered in the simultaneous treatment, the alloyed/compound coating should be thicker than that formed by SMAT with subsequent alloying treatment. Furthermore, the diffusive transport of species and phase transitions might be significantly promoted during SPD, which usually triggers the states equivalent to higher temperatures [88]. For example, Straumal et al. [89] demonstrated that high pressure torsion at room temperature produced a solid solution with a certain steady-state concentration equal to the solubility limit at an effective temperature of 680 °C in Cu–Ag alloys with different initial states. This will also contribute to the promoted surface alloying kinetics during simultaneous treatment, and might even result in the formation of metastable phases. However, deficiencies of such a treatment mainly come from technical difficulties in achieving simultaneous mechanical and alloying processes at elevated temperatures.

**Duplex surface alloying processes:** For surface alloying processes such as chromizing and aluminizing, temperatures as high as ≈1000 °C are conventionally required to achieved a satisfying (in terms of thickness and phase constitutions) alloyed/compound surface layer on steels. The formation kinetics of an alloyed layer might be significantly promoted by introducing a GNS surface layer. However, the formed alloyed layer was typically only on the micrometer scale in thickness at lower temperatures, even though a higher ratio was achieved by comparing the thickness of the alloyed layer on the GNS sample with that on the CG counterpart sample. A further increased processing temperature decreased the ratio or even reduce the thickness value due to the instability of nanostructures at higher temperatures, as mentioned previously. In addition, phase constitutions in compound layers formed at lower temperatures were sometimes different (sometimes not as satisfying) from conventional ones formed at higher temperatures. A duplex surface alloying processes, that is, a lower temperature (*T*_1_) process followed by a higher temperature (*T*_2_) process, have been developed to overcome these limitations [32–33,90]. The process at *T*_1_ allowed an effective diffusion of alloying elements and compound formation along GBs, while the nanostructures were relatively stable. The following process at *T*_2_ enhanced the growth (or phase transformation) kinetics of alloyed layers because the thermal stability in the surface layers was improved by the introduced alloying elements and/or compounds during the previous process.

**Figure 7:**
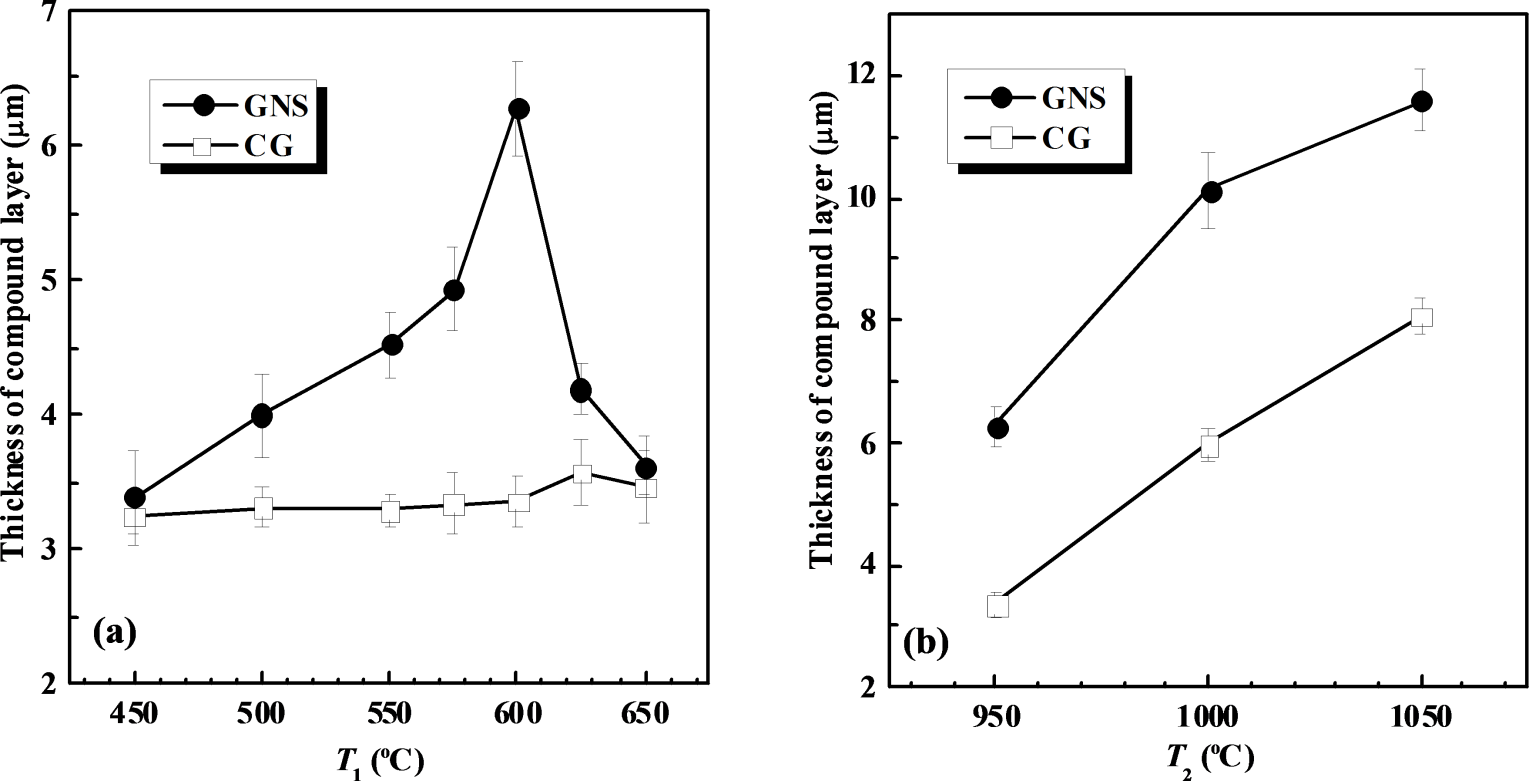
Effects of (a) *T*_1_ (with *T*_2_ = 950 °C for 120 min) and (b) *T*_2_ (with *T*_1_ = 600 °C for 120 min) on the compound layer thickness in the GNS and the CG H13 steel samples after duplex chromizing processes. Figure reproduced with permission from [33], copyright 2010 Elsevier.

The effects of both processes at *T*_1_ and at *T*_2_ on the formation of a chromized layer have been studied on a GNS H13 steel sample prepared by SMAT [33]. As shown in Figure 7, the thickness of the compound layer ((Cr,Fe)_2_N_1–x_ and (Cr,Fe)_23_C_6_ phases) increased gradually with increasing *T*_1_ before reaching a maximum at ≈600 °C and then decreased with a further increase in *T*_1_, when *T*_2_ was fixed at 950 °C for 120 min. In comparison, the compound layer formed on the CG sample remained approximately a constant thickness with increasing *T*_1_. When *T*_1_ was fixed at 600 °C for 120 min, a much thicker compound surface layer was formed on the GNS sample than on the CG sample after the chromizing process at *T*_2_ from 950 to 1050 °C. Subsequently, a duplex chromizing process at 600 °C followed by another at 1050 °C was employed to chromize the GNS sample. A chromized surface layer of ≈30 μm in thickness was formed, much thicker than that on the CG sample (≈10 μm) after the same chromizing process. And the wear resistance of the GNS sample after the duplex chromizing was significantly enhanced due to the higher surface hardness, as well as the graded distribution of composition, structure, and hardness.

In addition, a duplex aluminizing process including a packed aluminization process at 560 °C and a subsequent diffusion annealing treatment at 720 °C was developed for the P92 ferritic–martensitic (F-M) steel with a preformed GNS surface layer [90]. A lower aluminum content aluminide (AlFe and α-(FeAl)) surface layer of ≈17 μm in thickness was produced on the F-M steel. Experiments showed that the oxidation resistance of the F-M steel in steam was significantly promoted at higher temperatures. In comparison with the brittle Al-rich aluminide (mostly Al_5_Fe_2_) surface layer formed on the CG sample after the same duplex process, the lower content Al aluminide layer formed on the GNS sample is more resistant to cracking and has better adhesion to the substrate [91].

#### Other diffusion-related processes

The coating/substrate interface is usually the most vulnerable part in a coated component due to the lower bonding strength between them. Some experiments showed that the bonding property of a coating on a substrate might be improved by preforming a GNS surface layer on the substrate. This was mostly due to the promoted atomic diffusion kinetics in the GNS layer, which strengthened the metallurgical bonding across the interface. For example, the interdiffusion distance between the elements of a cold-sprayed Zn–Al coating and a GNS interstitial-free steel substrate was significantly increased in the as-sprayed state, resulting in an increment of ≈30% in the bonding strength of the coating on the substrate [92]. In addition, a higher film cohesion strength and better wear resistance have been achieved on the 304 stainless steel with a preformed GNS surface layer after being sputter-deposited a DLC coating, in comparison with that of the DLC-coated CG sample [93].

A GNS surface layer has also been employed to facilitate the formation of nanoporous titania on the commercial pure Ti [94–95], which is used as an important material in human body implantation. After immersion in the H_2_O_2_ solution for 24 h at room temperature, crystallized titania with the nanoporous structure was synthesized on the GNS Ti plate, while only interfacial corrosion was observed on the CG sample. By studying the structure and surface morphology evolution of the GNS sample in the H_2_O_2_ solution, Wen et al. [95] revealed that the accelerated formation of nanoporous structure was mostly due to the higher chemical reactivity of the GNS Ti, which promoted the decomposition of H_2_O_2_ and the formation of amorphous titania. Furthermore, the formed titania showed an increased crystallinity and retained the nanoporous structure even after calcination at 600 °C [94]. These works indicated the possibility to improve the bioactivity of titanium bone implants and to accelerate osseointegration by introducing a preformed GNS surface layer [95].

**Figure 8:**
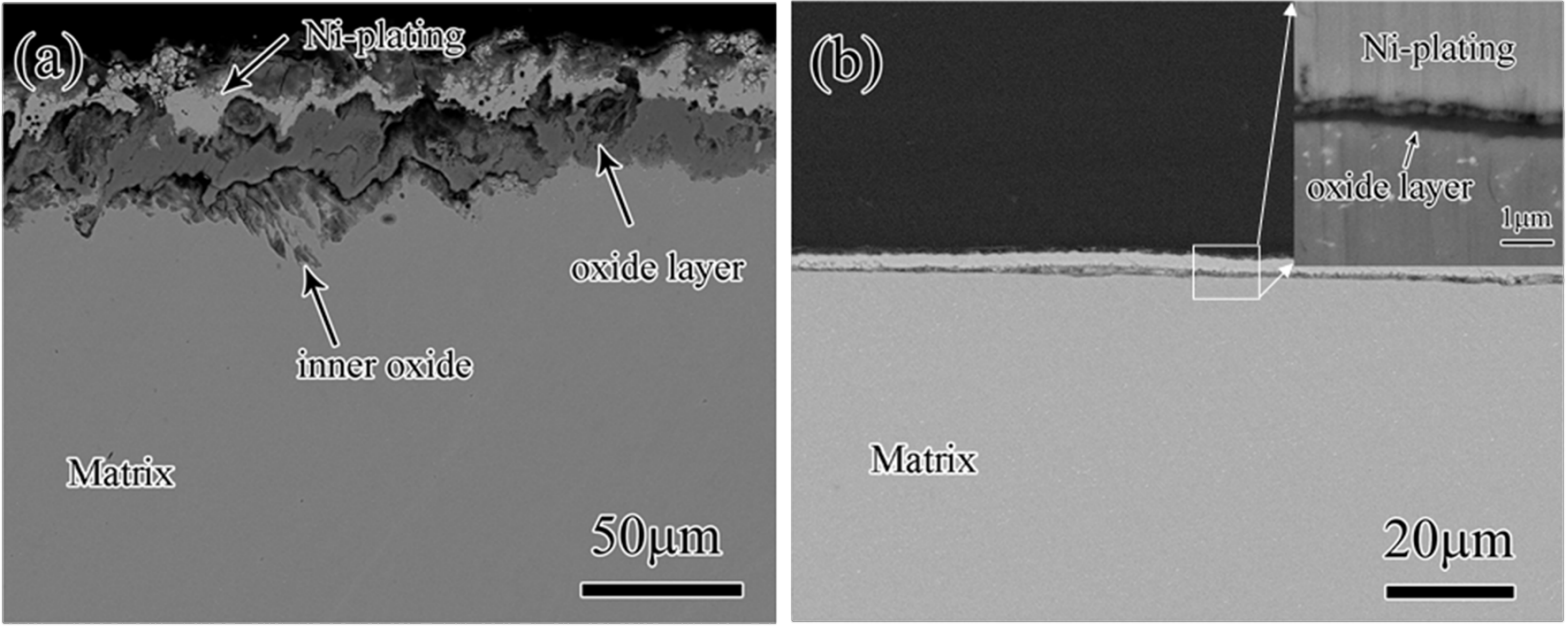
Typical cross-sectional morphologies of (a) the CG and (b) the GNS 9Cr2WVTa F-M steel samples oxidized at 600 °C for 510 h in air. Insert in (b) shows a magnified image of the boxed region. Figure reproduced with permission from [96], copyright 2016 Elsevier.

Different from the phenomena that thicker alloyed layers commonly form on GNS materials, a much thinner oxide layer was observed on a GNS 9Cr2WVTa F-M steel, i.e., the oxidation resistance of the steel was improved by the preformed GNS surface layer [96]. As shown in Figure 8, a compact oxide layer with the thickness of ≈0.5 μm was formed on the GNS sample after oxidation at 600 °C for 510 h in air. In comparison, it was within 8–50 μm in thickness and with various cracks on the oxidized CG sample. Further comparisons of composition and phase distributions indicated that the oxide scale was enriched with Mn and contained a large amount of Mn_2_O_3_ phase on the GNS sample, while no Mn enrichment was observed and the oxide scale was mainly composed of (Fe_0.6_Cr_0.4_)_2_O_3_ phase on the CG sample. Analysis confirmed that the markedly improved oxidation resistance of the GNS sample mostly resulted from the quicker formation of the Mn-enriched oxide scale. While the Mn oxide (i.e., Mn_2_O_3_) possessed the most negative free-energy among all possible oxides from 9Cr2WVTa steel, its formation was accelerated by the higher diffusivities in the GNS surface layer. In addition, the higher binding energy between Mn and vacancies, of which the concentration was much larger in the GNS sample than in the CG sample, also contributed to the quicker formation of Mn-enriched oxide scale [45,96–97].

## Conclusion

Due to the importance of understanding the diffusion and surface alloying behavior of GNS metals, interfacial diffusion and reactive diffusion have been systematically studied on this kind of material and summarized in this review. It was confirmed that interfacial diffusivities in GNS layers are typically 3–5 orders of magnitude higher than those along GBs in CG counterparts. This was mainly associated with a higher energy state of GBs induced by the plastic deformation with high strain and strain rates. Meanwhile, the chemical diffusion and reaction kinetics have been promoted in GNS surface layers, in which numerous GBs accelerate atomic diffusion and increase the nucleation and growth rates of formed compounds. The extra stored free energy at GBs also thermodynamically facilitate the formation of compounds in GNS surface layers.

Benefitting from the promoted diffusion and reaction kinetics, different surface alloying treatments have been achieved at much lower temperatures on materials with a preformed GNS surface layer than those of the conventional treatments on CG samples, such as nitriding, boronizing, and aluminizing. In addition, some other diffusion-related properties, such as bonding strength of coating on substrate, bioactivity of Ti and Ti alloys, and oxidation resistance of F-M steels, have also been enhanced by GNS surface layers. By applying a duplex approach including a lower temperature step and a subsequent higher temperature step, chromizing and aluminizing treatments were further modified to obtain an alloyed (or compound) surface layer with larger thickness and better properties.

Studies of diffusion behavior might shed light on the structural characteristics and properties of internal interfaces in nanostructured materials. Novel microstructures such as LAGB-dominant interfaces and nanolaminates have been synthesized in GNS metals recently [98–99]. They showed unique performance, for example, ultrahigh hardness accompanied by superior thermal stability. Studying the diffusion kinetics and relative behavior in GNS layers with such novel microstructures is expected to help understand their unique features and provide insights on tailoring the microstructure for better performance.

Combined with surface alloying, a graded transition in composition and/or phase constitution is formed in a GNS surface layer. Besides the enhanced properties, novel and unique properties of such a material with graded distribution of microstructure, composition, and phase are expected to be further explored. For example, a synergic improvement in strength, ductility, and resistance to wear and corrosion might be expected, while the distribution of microstructure, composition, and phase can be controlled within broader ranges due to the promoted diffusion kinetics in the preformed GNS layers.
